# Biosynthesis of Polyunsaturated Fatty Acids in Sea Urchins: Molecular and Functional Characterisation of Three Fatty Acyl Desaturases from *Paracentrotus lividus* (Lamark 1816)

**DOI:** 10.1371/journal.pone.0169374

**Published:** 2017-01-04

**Authors:** Naoki Kabeya, Alicia Sanz-Jorquera, Stefano Carboni, Andrew Davie, Angela Oboh, Oscar Monroig

**Affiliations:** Institute of Aquaculture, Faculty of Natural Sciences, University of Stirling, Stirling, Scotland, United Kingdom; Universiti Sains Malaysia, MALAYSIA

## Abstract

Sea urchins are broadly recognised as a delicacy and their quality as food for humans is highly influenced by their diet. Lipids in general and the long-chain polyunsaturated fatty acids (LC-PUFA) in particular, are essential nutrients that determine not only the nutritional value of sea urchins but also guarantee normal growth and reproduction in captivity. The contribution of endogenous production (biosynthesis) of LC-PUFA in sea urchins remained unknown. Using *Paracentrotus lividus* as our model species, we aimed to characterise both molecularly and functionally the repertoire of fatty acyl desaturases (Fads), key enzymes in the biosynthesis of LC-PUFA, in sea urchins. Three Fads, namely FadsA, FadsC1 and FadsC2, were characterised. The phylogenetic analyses suggested that the repertoire of Fads within the Echinodermata phylum varies among classes. On one hand, orthologues of the *P*. *lividus* FadsA were found in other echinoderm classes including starfishes, brittle stars and sea cucumbers, thus suggesting that this desaturase is virtually present in all echinoderms. Contrarily, the FadsC appears to be sea urchin-specific desaturase. Finally, a further desaturase termed as FadsB exists in starfishes, brittle stars and sea cucumbers, but appears to be missing in sea urchins. The functional characterisation of the *P*. *lividus* Fads confirmed that the FadsA was a Δ5 desaturase with activity towards saturated and polyunsaturated fatty acids (FA). Moreover, our experiments confirmed that FadsA plays a role in the biosynthesis of non-methylene interrupted FA, a group of compounds typically found in marine invertebrates. On the other hand, both FadsC desaturases from *P*. *lividus* showed Δ8 activity. The present results demonstrate that *P*. *lividus* possesses desaturases that account for all the desaturation reactions required to biosynthesis the physiological essential eicosapentaenoic and arachidonic acids through the so-called “Δ8 pathway”.

## Introduction

**Fig 1:**
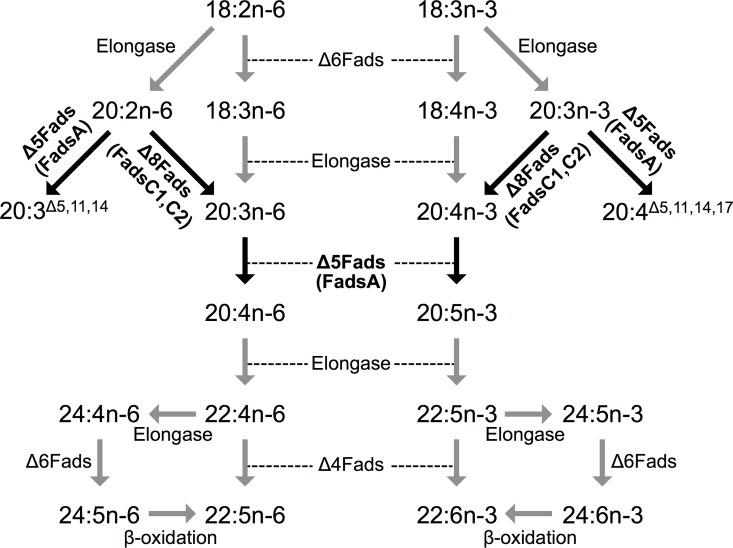
Biosynthetic pathways of LC-PUFA from C_18_ PUFA precursors accepted in vertebrates. Reactions catalysed by fatty acyl desaturases are designated as “Δx” (Δ6, Δ5, Δ4 and Δ8), whereas elongation reactions are indicated as “elongase”.

Long-chain (C_20-22_) polyunsaturated fatty acids (LC-PUFA) have been identified as essential components of biomembranes of all cells and tissues, and have important roles in growth and ontogenesis, particularly in development of the nervous system [1–2]. In addition, LC-PUFA have also key roles in inflammatory response and consequently in metabolic disorders, cardiovascular conditions and neurological diseases [3–5]. It is well known that vertebrates have some ability to biosynthesise LC-PUFA but are unable to endogenously produce their precursors, more specifically the C_18_ polyunsaturated fatty acids (PUFA) linoleic acid (18:2n-6, LA) and α-linolenic acid (18:3n-3, ALA). Consequently, diets for vertebrates must supply the C_18_ PUFA that are subsequently converted into the physiological important LC-PUFA, namely arachidonic acid (20:4n-6, ARA), eicosapentaenoic acid (20:5n-3, EPA) and docosahexaenoic acid (22:6n-3, DHA) [6]. The vertebrate LC-PUFA biosynthetic pathways consist of sequential reactions converting the dietary essential C_18_ PUFA into C_20-22_ LC-PUFA through the action of enzymes termed as fatty acyl desaturases (Fads) and elongation of very long-chain fatty acids proteins (Elovl) (Fig 1). Fads are key enzymes that mediate the introduction of an unsaturation (double bond) into a fatty acyl chain, while Elovl catalyse the condensation reaction within the elongation pathway resulting in the addition of two carbons into the fatty acid (FA) substrate [7–8].

Unlike vertebrates, the biosynthetic pathways of LC-PUFA in marine invertebrates remain poorly understood [9]. Pioneer studies conducted in the common octopus *Octopus vulgaris* [10,11] and thereafter in the cephalopod *Sepia officinalis* [12], the gastropod *Haliotis discus hannai* [13] and the bivalve *Chlamys nobilis* [14–16] have confirmed that molluscs and potentially other marine invertebrates possess Fads and Elovl enzymes involved in the LC-PUFA biosynthesis. Additionally, the ability to synthesise non-methylene interrupted (NMI) FA, a group of PUFA with particular double bond distribution [17], was observed in several Fads enzymes from marine invertebrate species [10,12].

A recent study on Fads and Elovl repertoire among several classes of molluscs including cephalopods, gastropods and bivalves [18] highlighted that lineage-specific gene duplication events accounted for the presence of one or more Fads copies among the mollusc classes under investigation. These results further evidenced that the evolutionary history of genes involved in the LC-PUFA biosynthetic pathways of invertebrates, particularly the Fads-like desaturases, might differ markedly from those of vertebrates [6,12,19]. Clearly, marine invertebrates emerge as promising sources of LC-PUFA biosynthetic enzymes with potentially novel functionalities amenable for biotechnological production of n–3 oils [20].

Some invertebrate groups and specific species among them are becoming increasingly popular model species for comparative genomics and evolutionary developmental biology [21–23]. As a result, ever-increasing genomic data on certain species are becoming available. Among echinoderms, there exist genome projects developed for the sea urchin *Strongylocentrotus purpuratus* [24] and *Lytechinus variegatus* [25], and this offers us a unique opportunity to provide a comprehensive characterisation of key protein families involved in the biosynthesis of LC-PUFA in commercially interesting species such as *Paracentrotus lividus* (Lamark, 1816). *P*. *lividus* is a herbivorous sea urchin species that is commonly known as the Atlanto-Mediterranean sea urchin, due to its habitats comprising the sublittoral zone to 20 m in both the Mediterranean Sea [26] and throughout West coasts of continental Europe to Ireland and Scotland, UK [27]. Gonads from *P*. *lividus* are recognised as a high value seafood and delicacy [28], and thus *P*. *lividus* has been studied in different countries such as UK, Ireland and The Netherlands [29–31]. Recently comprehensive research has been conducted to optimise *P*. *lividus* culture, through assessing the nutritional requirements as a key aspect to fully develop a culture protocol for this species [32–35]. It has been reported that the growth and gonadal development are greatly affected by feed abundance and quality [36] and that adequate food supply during early development ensures optimal sexual maturation [31,33]. In addition, n-3 LC-PUFA such as EPA and DHA showed beneficial effect on early development of *P*. *lividus* [37]. Clearly, understanding the ability of *P*. *lividus* to endogenously produce essential LC-PUFA ensuring normal growth and reproduction is required to adequately formulate balanced diets. Therefore, it is important to elucidate the functions and substrate specificities of the endogenous enzymes that are responsible for the LC-PUFA biosynthesis. To the best of our knowledge, the specific Fads repertoire and functionalities of sea urchins remains to be investigated. Thus, the aim of this study was to identify the whole set of Fads-like desaturases found in *P*. *lividus* and characterise their function in yeast. The results will also provide valuable information on the evolution of the Fads family, and better understanding of LC-PUFA biosynthesis in sea urchin and, in extension, other echinoderms.

## Materials and Methods

### Molecular cloning and phylogenetic analysis of novel fatty acyl desaturases from *P*. *lividus*

Fresh gonad, intestine and tube feet samples were collected from a *P*. *lividus* specimen from Ardtoe Marine Research Facility (Scotland, UK), and conserved in RNAlater (Thermo Fisher Scientific, Waltham, MA, USA) until further use. The total RNA was extracted from the selected tissues (~100 mg) using TRI Reagent (Sigma-Aldrich, Dorset, UK) following the manufacturer’s instructions. Complementary DNA (cDNA) were synthesised from 1 μg of total RNA using High Capacity cDNA Reverse Transcription Kit (Thermo Fisher Scientific) following the manufacturer’s instructions.

In order to amplify the first fragment of *P*. *lividus* Fads cDNA, we retrieved several Fads-like sequences from expressed sequence tags (EST) information of *P*. *lividus* available in public databases. After alignment and phylogenetic analysis of obtained sequences, it was possible to distinguish two different Fads types. One of them closely related to several Fads genes characterised from other invertebrate species such as molluscs. Phylogenetic analysis performed by Surm and co-workers [18] indicated that this type of Fads formed a distinct cluster denoted as “clade A”, and consequently we termed the *P*. *lividus* Fads homologue as “FadsA”. On the other hand, we termed the other Fads-like sequence retrieved from the EST database search as “FadsC1”, to indicate it belongs to a cluster (“clade C”) different to Clades A or B reported in molluscs [18]. The first fragment of FadsA was amplified by PCR (Klear *Taq* polymerase, LGC, Teddington, UK) using the tube feet cDNA as template and the primers PLFAF and PLFAR, which were designed to anneal to a consensus sequence derived from EST (NCBI accession No. AM559332, AM220309, AM571147 and AM537908). The PCR amplification was performed with 35 cycles comprising denaturation for 20 s at 95°C, annealing for 20 s at 60°C and extension for 60 s at 72°C. In order to amplify the first fragment of *P*. *lividus* FadsC1, a forward primer PLFC1F (Table 1) was also designed to anneal to a consensus sequence derived from EST (NCBI accession No. AM569687, AM566789 and AM569285). Since the sequences obtained from EST databases were lacking most of the 3' region of putative coding sequence of FadsC1, we designed a reverse primer using consensus sequence derived from other sea urchin species in order to amplify a longer fragment. A reverse primer URCHFC1R (Table 1) was designed to anneal to a consensus sequence of FadsC1 homologues obtained from genomic and transcriptomic data of several sea urchin species (*S*. *purpuratus* NW_011995688, *Evechinus chloroticus* GAPB01052974, *L*. *variegatus* GAUR01019274, *Sphaerechinus granularis* GAVR01046553). The first fragment of FadsC1 was amplified from a mixture of the intestine and gonad cDNA by GoTaq® Colorless Master Mix (Promega, Madison, WI, USA). The PCR amplification was performed with 35 cycles comprising denaturation for 30 s at 95°C, annealing for 30 s at 55°C and extension for 60 s at 72°C. The amplified fragments were purified on agarose gels using Illustra^TM^ GFX^TM^ PCR DNA and Gel Band Purification Kit (GE Healthcare Life Sciences, Buckinghamshire, UK). The purified PCR products were then sequenced (GATC Biotech: DNA Sequencing and Bioinformatics, Konstanz, Germany).

**Table 1:** Sequences of the primer pairs used in the molecular cloning and functional characterisation in yeast of the *Paracentrotus lividus* fatty acyl desaturases (FadsC1, FadsA and FadsC2). Restriction sites for *Hin*dIII and *Xba*I are underlined (AAGCTT and TCTAGA, respectively).

Aim	Target	Primer	Primer sequence
First fragment	*FadsA*	PLFAF	5'-TCACGCAGTGGGCCAAGAGACA-3'
		PLFAR	5'-ACAGAAGAGGGGGTCCAATGAGGA-3'
	*FadsC1*	PLFC1F	5'-GCGTGAGTCATAACAAGCCA-3'
		URCHFC1R	5'-TAGCAAGATTGTGTCTCGGCAT-3'
5' RACE PCR	*FadsA*	PLFAR1	5'-GGAAGGTCCACCAACAGAATCCATA-3'
		PLFAR2	5'-TCTTTGGCGATCTGCCCAATGTGGA-3'
	*FadsC1*	PLFC1R1	5'-GCGCGGTGTCATCAAGGTT-3'
		PLFC1R2	5'-TTGACTCACTGGAGCGATGAC-3'
3' RACE PCR	*FadsA*	PLFAF1	5'-GCATGGCACACTACAGGCTCAGGT-3'
		PLFAF2	5'-TCATTGGACCCCCTCTTCTGTTTC-3'
	*FadsC1*	PLFC1F1	5'-TCTTCAGATGCATGCCACGTGTA-3'
		PLFC1F2	5'-ACCTGGAGTCTTCTCTTTTCATTG-3'
ORF cloning	*FadsA*	PLFAVF	5'-CCCAAGCTTACGATGGGTCTGGGAG-3'
		PLFAVR	5'-CCGTCTAGATTAGTCCGTTGAATACTGGT-3'
	*FadsC1*	PLFC1VF	5'-CCCAAGCTTACAATGTGGACGATTAGAGA-3'
		PLFC1VR	5'-CCGTCTAGACTACCCTACATAAGCTCCTA-3'
	*FadsC2*	PLFC2VF	5'-CCCAAGCTTACGATGTGCAAGAAGGAAGATCTCT-3'
		PLFC2VR	5'-CCGTCTAGATCAATGGTCGCCTGCAGGATCTA-3'

The DNA sequences of the first fragments of FadsA and FadsC1 cDNAs were used to design primers for 5' and 3' Rapid Amplification of cDNA Ends (RACE) (Table 1). The RACE cDNAs were synthesised using the SMART RACE kit (Takara Bio USA, Inc., Mountain View, CA, USA) from the tube feet RNA and the FirstChoice® RLM-RACE Kit (Thermo Fisher Scientific) from the intestine and gonad RNA. Regarding the RACE PCR for FadsA, all reactions including first and second (nested) rounds were carried out with 30 cycles comprising denaturation for 20 s at 95°C, annealing for 20 s at 60°C and extension for 180 s at 72°C using adequate primers (Table 1) and the adapter primers supplied with the kit. Regarding FadsC1, both first and nested 5' RACE were carried out with 35 cycles comprising denaturation for 30 s at 95°C, annealing for 30 s at 55°C and extension for 120 s at 72°C using adequate primer sets (Table 1) and the adapter primers. Both first and second round 3' RACE PCRs were carried out with 35 cycles comprising denaturation for 30 s at 95°C, annealing for 30 s at 58°C and extension for 90 s at 72°C using adequate primer sets (Table 1) and the adapter primers. The nested PCR products were purified and then sequenced as described above.

After cloning of the full-length cDNA of FadsA and FadsC1, *P*. *lividus* transcriptome shotgun assembly was released in NCBI (http://www.ncbi.nlm.nih.gov) and this allowed us to screen other possible Fads-like transcripts in *P*. *lividus*. Thus, we obtained full-length open reading frame (ORF) sequence of another Fads-like sequence from the assembly (accession no. GCZS01077556). Since preliminary phylogenetic analysis suggested this Fads was closely related to FadsC1, we termed this desaturase as “FadsC2”.

The deduced amino acid (aa) sequences of *P*. *lividus* desaturases FadsA, FadsC1 and FadsC2 were aligned with homologous Fads sequences from various species following ClustalW algorithm. The phylogenetic tree of deduced aa sequences was constructed using the neighbour-joining method [38] with confidence in the resulting tree branch topology measured by bootstrapping through 1,000 iterations. All the sequencing analyses were carried out using CLC Main Workbench 7 (CLC bio, Aarhus, Denmark).

### Functional characterisation of newly cloned Fads from *P*. *lividus*

The full-length ORF sequences of the *P*. *lividus* FadsA, FadsC1 and FadsC2 were amplified from the intestine cDNA using the high fidelity *Pfu* DNA polymerase (Promega). All the PCR amplifications were carried out with 35 cycles comprising denaturation for 30 s at 95°C, annealing for 30 s at 55°C and extension for 180 s at 72°C. The primer pairs contained restriction sites for *Hin*dIII (forward) and *Xba*I (reverse) for further cloning into the yeast expression vector pYES2 (Thermo Fisher Scientific). The PCR products were digested with *Hin*dIII and *Xba*I and then ligated into the similarly restricted pYES2 vector using T4 DNA ligase (Promega). Subsequently, the ligation reactions were transformed into competent *E*. *coli* JM109 cells (Promega). The plasmid constructs pYES2-FadsA, pYES2-FadsC1 and pYES2-FadsC2 were prepared (Gen Elute^TM^ Plasmid Miniprep Kit, Sigma-Aldrich) and sequenced prior being transformed into competent yeast INv*Sc*1 cells (Thermo Fisher Scientific) using *S*.*c*. EasyComp Transformation kit (Thermo Fisher Scientific). Selection of successful transformants and culture of transgenic yeast were performed as described in previous studies [10,11].

One single yeast colony transformed with either the *P*. *lividus* FadsA, FadsC1 or FadsC2 was used in each functional assay [10,11]. In order to investigate the ability of the *P*. *lividus* FadsA, FadsC1 and FadsC2 to desaturate PUFA, the transgenic yeast were grown in the presence of Δ6 (18:3n-3 and 18:2n-6), Δ8 (20:3n-3 and 20:2n-6), Δ5 (20:4n-3 and 20:3n-6) and Δ4 (22:5n-3 and 22:4n-6) substrates. Additionally, the transgenic yeast were also grown in the presence of 20:1n-9 to assess the ability of the *P*. *lividus* desaturases to biosynthesise NMI FAs. All FA substrates (>98–99% pure) used for the functional characterisation assays were obtained from Nu-Chek Prep, Inc. (Elysian, MN, USA), except 20:4n-3 that was purchased from Cayman Chemical Co. (Ann Arbor, USA). Since the newly cloned *P*. *lividus* Fads could potentially operate towards yeast endogenous saturated or monounsaturated FA, we further compared the FA profiles of yeast transformed with either pYES2-FadsA, pYES2-FadsC1 or pYES2-FadsC2 with that of yeast transformed with the empty pYES2 (control), and grown in all cases in the absence of exogenously added PUFA substrates. Each PUFA substrate was supplied to the yeast cultures in concentrations corresponding to 0.5 (C_18_), 0.75 (C_20_) and 1 mM (C_22_) as uptake efficiency decreases with increasing chain length [12].

### Fatty acid analysis

Total lipids were extracted from yeast samples according to [39] and modifications as described in [10]. Fatty acid methyl esters (FAME) were prepared from total lipids as described previously [40]. FAME were quantified and identified using a Fisons GC-8160 (Thermo Fisher Scientific) gas chromatograph equipped with a 60 m x 0.32 mm i.d. x 0.25 μm ZB-wax column (Phenomenex, Cheshire, UK) and flame ionisation detector [41]. The desaturase conversions from exogenously added PUFA substrates were calculated from the fraction of FA substrate transformed to desaturase FA products as [product areas / (product areas + substrate area)] x 100. In order to determine the double bond positions of unusual FAs, the fatty acid 4,4-dimethyloxazoline (DMOX) derivatives were prepared from FAMEs. Briefly, the dried-up FAME samples were incubated over-night with 2-amino-2-methyl-1-propanol at 150°C. After cooling, the DMOX derivatives were extracted by adding 5 mL of distilled water and 5 mL diethyl ether-isohexane (1:1, v/v). The organic phase was transferred to fresh tubes and then evaporated under the stream of oxygen-free nitrogen. Subsequently, the dried-up DMOX samples were resuspended with isohexane. The DMOX derivatives were identified and quantified using a gas chromatograph (GC8035) equipped with a 30 m x 0.32 mm i.d. x 0.25 μm ZB-wax column (Phenomenex) and coupled to an MD800 mass spectrometer (ThermoFisher Scientific).

## Results

### *P*. *lividus* Fads sequences and phylogenetics

**Fig 2:**
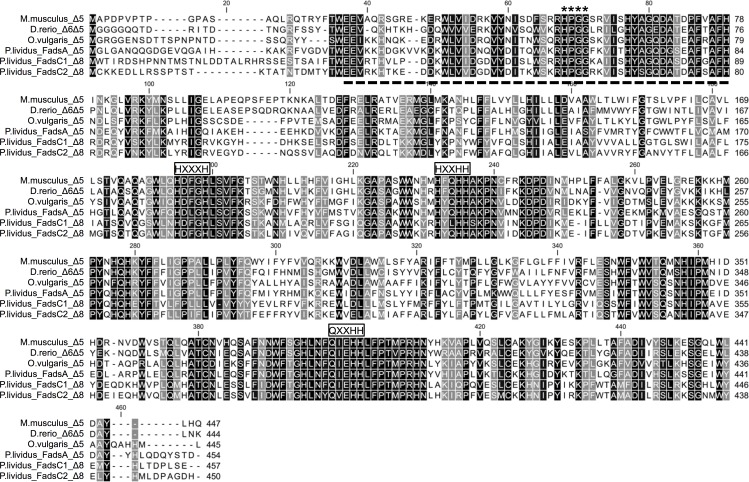
Comparison of the deduced amino acid sequence of the *Paracentrotus lividus* desaturases with those of mice (*Mus musculus*), zebrafish (*Danio rerio*) and common octopus (*Octopus vulgaris*). Identical sequences are shaded black. All the Fads sequence from *P*. *lividus* contained typically conserved regions in members of the front-end desaturase family, including three histidine boxes (HXXXH, HXXHH and QXXHH), a putative cytochrome b5-like region (marked as a dotted line) and a heme-binding motif (HPGG).

**Fig 3:**
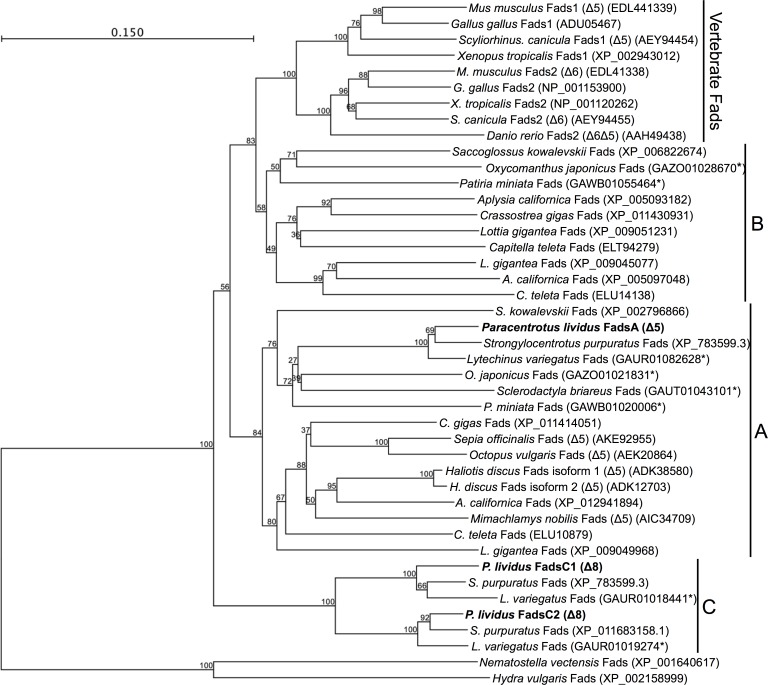
Phylogenetic tree comparing the deduced amino acid (aa) sequence of the *Paracentrotus lividus* fatty acyl desaturases (Fads) with other Fads-like sequences from different organisms (vertebrates and invertebrates). FadsA, FadsC1 and FadsC2 are highlighted in black. The tree was constructed using the Neighbour Joining method [38]. The horizontal branch length is proportional to aa substitution rate per site. The numbers represent the frequencies (%) with which the tree topology presented was replicated after 1,000 iterations. Asterisks denote deduced aa sequences derived from transcriptome shotgun assembly (TSA) from several echinoderm species.

The ORF of the newly cloned *P*. *lividus* FadsA (GenBank accession number KY216020), FadsC1 (KY216021) and FadsC2 (KY216022) consisted of 1365 bp, 1374 bp and 1353 bp, respectively, encoding putative proteins of 454 aa, 457 aa and 450 aa, respectively. All three desaturases had representative domains of the “front-end” desaturases, including three histidine boxes (HXXXH, HXXHH and QXXHH), a putative cytochrome b5-like region and a heme-binding motif (HPGG) (Fig 2). The aa identity of *P*. *lividus* desaturases varied between FadsA and FadsC1 (45% identical) or FadsC2 (45%), and between FadsC1 and FadsC2 (64%). Phylogenetic analysis revealed that each *P*. *lividus* Fads protein was closely related to orthologues found in the sea urchin species *S*. *purpuratus* and *L*. *variegatus* (Fig 3). Compared with the three Fads-like desaturases found in the genome of *S*. *purpuratus*, the three *P*. *lividus* desaturases showed high aa sequence identity, namely 81% (FadsA *vs* XP_783599.3), 85% (FadsC1 *vs* XP_783599.3), and 85% (FadsC2 *vs* XP_011683158.1). Moreover, FadsA clustered with Fads from several molluscs that have all been functionally characterised as Δ5 desaturases (Fig 3, clade A) [10,12–14]. In contrast to FadsA, the desaturases FadsC1, FadsC2 and Fads-like sequences from other sea urchin species uniquely formed a distinct clade from other Fads (Fig 3, clade C). Interestingly, Fads-like sequences from some echinoderm species of starfish and sea lilies formed a distinct group denoted as “clade B” in Fig 3. Among all three types of Fads identified in echinoderms, Fads within clade B (FadsB) are the most closely related to vertebrate Fads, although this orthologue appears to be absent in the genomes of *S*. *purpuratus* and *L*. *variegatus* and was not found either in *P*. *lividus* transcriptomic databases. These results suggest that clade B desaturases do not exist in echinoids.

### Functional characterisation of the *P*. *lividus* Fads in yeast

**Fig 4:**
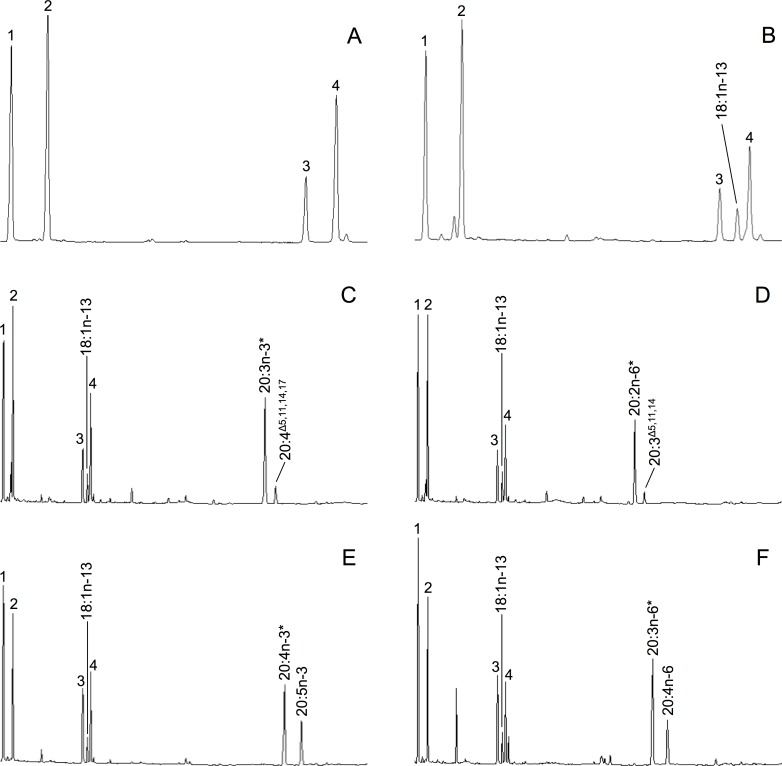
Functional characterisation of FadsA from *Paracentrotus lividus* in transformed yeast *Saccharomyces cerevisiae*. Additional 18:1n-13 peak was observed in all the yeast transformed with pYES2-FadsA (B–F) compared with the yeast transformed with empty pYES2 vector (A). The yeast transformed with pYES2-FadsA were also grown in the presence of fatty acid (FA) substrates (indicated by asterisk), namely 20:3n-3 (C), 20:2n-6 (D), 20:4n-3 (E) and 20:3n-6 (F). Desaturation products are indicated accordingly in each panel. Peaks 1–4 in all panels are the main endogenous FA of *S*. *cerevisiae*, namely 16:0 (1), 16:1 isomers (2), 18:0 (3), 18:1n-9 (4). Vertical axis, FID response; horizontal axis, retention time.

The activity of the *P*. *lividus* desaturases (FadsA, FadsC1 and FadsC2) was determined by expression of their ORF in yeast *S*. *cerevisiae* that were grown in presence of potential FA substrates. The FA profiles of yeast transformed with the empty vector (control), typically showed the endogenous *S*. *cerevisiae* FA, namely 16:0, 16:1 isomers (16:1n-9 and 16:1n-7), 18:0, 18:1 isomers (18:1n-9 and 18:1n-7) (Fig 4A). When additional PUFA substrates were exogenously supplied, these remained unmodified by control yeast confirming that the *S*. *cerevisiae* yeast strain INv*Sc*1 lack Δ4, Δ5, Δ6 and Δ8 desaturase activities [42].

The capability of *P*. *lividus* Fads to desaturate saturated FA was studied by comparing the FA profiles of yeast transformed with pYES2-FadsA, pYES2-FadsC1 and pYES2-FadsC2, with that of control yeast. The FA profiles from yeast transformed with either pYES2-FadsC1 or pYES2-FadsC2 and grown in the absence of exogenously added PUFA substrates, showed no additional peaks compared to controls. However, the FA profiles of yeast expressing the *P*. *lividus* FadsA had an additional peak corresponding to the Δ5-desaturated monoene 18:1n-13 (Fig 4B). The MS peak of a DMOX derivative of 18:1n-13 contained a diagnostic ion at m/z = 153, which shows a double bond at the Δ5 position (S1A Fig) and also matched the spectra presented in the AOCS lipid library website (http://lipidlibrary.aocs.org). Such Δ5 desaturation product from 18:0 occurred in all samples of yeast transformed with pYES2-FadsA, regardless the exogenously added PUFA substrate (data not shown). Conversion of 18:0 into 18:1n-13 was 32% (Table 2).

**Table 2:** Substrate conversions of yeast *Saccharomyces cerevisiae* transformed with pYES2 containing the open reading frame (ORF) of the *Paracentrotus lividus* desaturases (FadsC1, FadsA and FadsC2). Results are expressed as a percentage of total fatty acid (FA) substrate converted to desaturated products, with the corresponding activity (Δx) detected also shown. FA are designated using the ‘n-‘ nomenclature, except for the non-methylene interrupted FA produced from 20:3n-3 and 20:2n-6 where the ‘Δ’ nomenclature is used.

FA substrates	Product	Conversion rate (%)			Activity
		FadsA	FadsC1	FadsC2	
Saturate					
18:0	18:1n-13	32	nd	nd	Δ5
Polyunsaturates					
18:3n-3	18:4n-3	nd	nd	nd	Δ6
18:2n-6	18:3n-6	nd	nd	nd	Δ6
20:3n-3	20:4n-3	nd	4	42	Δ8
	20:4^Δ5,11,14,17^	13	nd	nd	Δ5
20:2n-6	20:3n-6	nd	2	38	Δ8
	20:3^Δ5,11,14^	12	nd	nd	Δ5
20:4n-3	20:5n-3	37	nd	nd	Δ5
20:3n-6	20:4n-6	30	nd	nd	Δ5
22:5n-3	22:6n-3	nd	nd	nd	Δ4
22:4n-6	22:5n-6	nd	nd	nd	Δ4

nd, not detected

**Fig 5:**
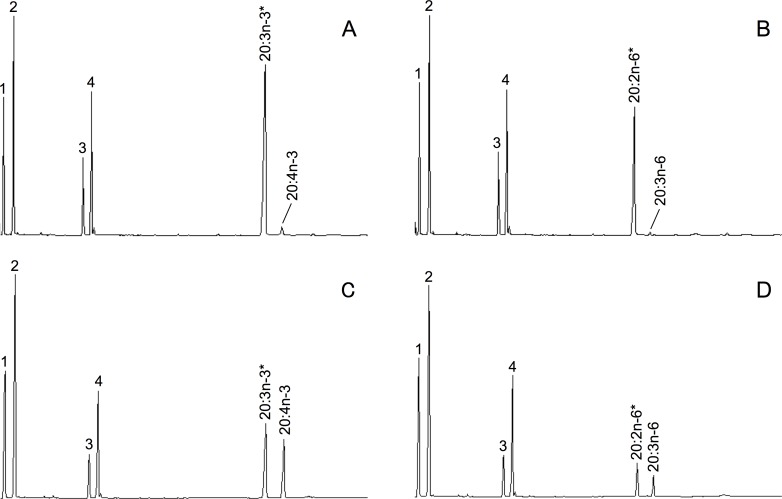
Functional characterisation of FadsC1 and FadsC2 from *Paracentrotus lividus* in transformed yeast *S*. *cerevisiae*. The fatty acid (FA) profiles of yeast transformed with pYES2-FadsC1 (A, B) and pYES2-FadsC2 (C, D) were determined after they were grown in the presence of FA substrates (indicated by asterisk), namely 20:3n-3 (A, C) and 20:2n-6 (B, D). Moreover, desaturation products are indicated accordingly in each panel. Peaks 1–4 in all panels are the main endogenous FA of *S*. *cerevisiae*, namely 16:0 (1), 16:1 isomers (2), 18:0 (3), 18:1n-9 (4). Vertical axis, FID response; horizontal axis, retention time.

The function of the *P*. *lividus* FadsA, FadsC1 and FadsC2 in LC-PUFA biosynthetic pathways was investigated by growing transgenic yeast in the presence of PUFA precursors including 18:3n-3, 18:2n-6, 20:3n-3, 20:2n-6, 20:4n-3, 20:3n-6, 22:5n-3 and 22:4n-6. Transgenic yeast expressing the *P*. *lividus* FadsA were able to transform 20:4n-3 and 20:3n-6 into the corresponding Δ5 products, namely 20:5n-3 (EPA) and 20:4n-6 (ARA), respectively (Fig 4E and 4F). In addition, transgenic yeast carrying the *P*. *lividus* FadsA were able to desaturate 20:3n-3 and 20:2n-6 into the NMI FA 20:4^Δ5,11,14,17^ and 20:3^Δ5,11,14^, respectively (Fig 4C and 4D). The MS of the peaks identified as 20:4^Δ5,11,14,17^ and 20:3^Δ5,11,14^ contained a diagnostic ion for Δ5 desaturation at m/z = 153 (S1B and S1C Fig). Thus, 20:4^Δ5,11,14,17^-DMOX showed the characteristic gaps of 12 atomic mass units (amu) between m/z = 222 and 234, 262 and 274, and 302 and 314, which indicate the presence of the double bonds at Δ11, Δ14 and Δ17 position, respectively (S1B Fig). The MS profile of 20:3^Δ5,11,14^-DMOX also showed 12 amu between 222 and 234, and 262 and 274, which correspond to the double bonds at Δ11 and Δ14 position, respectively (S1C Fig). No activity towards 18:3n-3, 18:2n-6, 22:5n-3, 22:4n-6 was detected in the FA profiles (Table 2). These results confirmed that FadsA is a Δ5 desaturase. On the other hand, in yeast expressing the *P*. *lividus* FadsC1 and FadsC2, additional peaks identified as 20:4n-3 and 20:3n-6 were detected in yeast grown in the presence of 20:3n-3 and 20:2n-6, respectively (Fig 5A, 5B, 5C and 5D). These conversions clearly showed that both FadsC1 and FadsC2 are Δ8 desaturases. Neither FadsC1 nor FadsC2 showed any activity towards 18:3n-3, 18:2n-6, 20:4n-3, 20:3n-6, 22:5n-3 and 22:4n-6. In addition, no activity towards 20:1n–9 was observed in any of the three Fads cloned from *P*. *lividus*.

## Discussion

Sea urchins are regarded as a delicacy in many countries; their gonads are commonly consumed as a raw product. As a result of their high economical value, the sea urchin culture industry aims to produce individuals with roe having the taste, appearance and the nutritional value as close as possible to those of wild specimens. Previous studies suggested that dietary lipids partly determined the lipid contents of *P*. *lividus* tissues [43] but it remained unclear whether endogenous biosynthesis of lipids and fatty acid can also contribute to the overall nutritional value. In the present study, we cloned and functionally characterised three distinct desaturases, namely FadsA, FadsC1 and FadsC2, with a role in the biosynthetic pathways of LC-PUFA in *P*. *lividus*.

The deduced aa sequences of the three desaturases isolated from *P*. *lividus* indicated they were all front-end desaturases, enzymes that introduce double bonds between a pre-existing unsaturation and the carboxyl end of the fatty acyl chain, in the position Δx from the carboxyl group [44]. The cytochrome b5 domain, heme–binding motif (HPGG) and three histidine boxes (HXXXH, HXXHH and QXXHH), which are typical features of front-end desaturases [6,8,44], were found in all desaturase sequences obtained from *P*. *lividus*. In spite of sharing common features though, distinctive sequence domains among Fads from sea urchins and other Echinodermata classes became obvious from our phylogenetic analysis. Clearly, our phylogenetic analysis confirms that the repertoire of Fads within the Echinodermata phylum varies among classes, similarly as reported for molluscs [18]. On one hand, the FadsA, confirmed be a Δ5 desaturase in *P*. *lividus*, is found in other echinoderm classes including starfish (*Patiria miniata*, Asteroidea), brittle stars (*Ophiothrix spiculata*, Ophiuroidea) and sea cucumbers (*Parastichopus parvimensis*, Holothuroidea), thus suggesting that this desaturase is virtually present in all echinoderms. On the other hand, the FadsC appears to be a Echinoidea-specific desaturase and, indeed, two distinct genes have been identified in *P*. *lividus* (FadsC1 and FadsC2) and genomes of *S*. *purpuratus* and *L*. *variegatus*. Interestingly, neither starfishes, brittle stars nor sea cucumbers appear to possess FadsC-like desaturases according to genomic information available from NCBI (accession no. GCA_000285935, GCA_000969725, GCA_000934455, respectively). Finally, the latter echinoderm classes but not sea urchins (Echinoidea), possess a desaturase herein denoted as FadsB. Importantly, our phylogenetic analysis suggested that the Echinodermata FadsB is the most closely related Fads-like desaturase to the vertebrate orthologues, and thus arises as an interesting protein to elucidate the evolutionary divergence of substrate specificity among metazoan Fads.

The differences in the amino acid sequences of the sea urchin Fads highlighted in the phylogenetic tree were further reflected in their regioselectivity. Thus the functional analyses in yeast confirmed that both FadsC1 and FadsC2 are Δ8 desaturases, a regioselectivity previously reported in a Fads from the scallop *Chlamys nobilis* [15]. Additionally, the *P*. *lividus* FadsA was confirmed to be a Δ5 desaturase, a desaturase ability reported in a variety of molluscs [10,12–14]. The present results demonstrate that *P*. *lividus* possesses desaturases that account for all the desaturation reactions required to convert the C_18_ PUFA precursors ALA (18:3n-3) and LA (18:2n-6) into the physiologically important EPA (20:5n-3) and ARA (20:4n-6), respectively, through the so-called “Δ8 pathway” [6] (Fig 1). These results are consistent with the fact that the two most abundant LC-PUFA in *P*. *lividus* lipids are usually EPA and ARA [45, 46], and can partly explain why high contents of ARA were found in the gonads of *P*. *lividus* individuals fed diets with low ARA content [45]. Similarly to the mollusc desaturases, the *P*. *lividus* FadsA also showed the capability to desaturate 18:0 into 18:1n-13 (18:1^Δ5^) but, in contrast to molluscs, the *P*. *lividus* FadsA was not able to desaturate 16:0 to 16:1n-11 (16:1^Δ5^) [10,12,14]. While dietary origin cannot be ruled out, the herein reported activity of the *P*. *lividus* FadsA is consistent with the presence of 18:1n-13 reported in several sea urchin species [47,48]. Overall, the functional analysis of the *P*. *lividus* FadsA clearly demonstrated that this enzyme is a Δ5 desaturase that can operate towards both saturated FA and PUFA substrates. Since the *P*. *lividus* FadsA clustered together with several mollusc Δ5 Fads in the phylogenetic tree (Clade A in Fig 3), it can be speculated that all Fads belonging to Clade A have Δ5 desaturase activity. Moreover, the FadsA appears to play a role in the biosynthesis of NMI FA in *P*. *lividus*.

The presence of NMI FA in sea urchins has been confirmed in previous studies [17,37,46–48,49–51], and biosynthetic pathways for certain NMI FA have even been postulated for *Strongylocentrotus droebachiensis* [52]. For instance, the biosynthesis of 20:2^Δ5,11^ and 20:2^Δ5,13^ has been hypothesised to proceed through Δ5 desaturation of 20:1n-9 (20:1^Δ11^) and 20:1n-7 (20:1^Δ13^), respectively [52]. In order to elucidate the molecular mechanisms underlying the biosynthesis of NMI FA in *P*. *lividus*, we incubated transgenic yeast expressing the newly cloned *P*. *lividus* Fads in the presence of 20:1n-9 (20:1^Δ11^). Our GC-MS analyses confirmed that no desaturation product was detected for any of the three Fads desaturases characterised from *P*. *lividus*. These results suggest that 20:2^Δ5,11^ and potentially other NMI FA reported in *P*. *lividus* tissues have a dietary origin rather than endogenous synthesis. Nevertheless, the *P*. *lividus* FadsA showed the ability to biosynthesise the NMI FAs 20:4^Δ5,11,14,17^ and 20:3^Δ5,11,14^ from the precursors 20:3n-3 (20:3^Δ11,14,17^) and 20:2n-6 (20:2^Δ11,14^), respectively. Similar functions have been also reported in Fads-like desaturases from common octopus, common cuttlefish and scallop [10,12,14]. Indeed these NMI FAs have been identified in several sea urchin species [47,48] and thus FadsA arises as the most likely candidate enzyme accounting for the biosynthesis of such NMI FA *in vivo*.

In conclusion, it has been demonstrated that *P*. *lividus* possess three fatty acyl desaturases with Δ5 desaturase (FadsA) and Δ8 desaturase (FadsC1 and FadsC2) activities. Moreover, we have provided evidence confirming that the *P*. *lividus* FadsA is involved in the biosynthesis of NMI FA, a role that could not be demonstrated for the FadsC desaturases. Our phylogenetic analysis revealed that the repertoire of Fads varies among echinoderm classes. FadsA desaturases can be found virtually in all Echinodermata classes, whereas other Fads-like desaturases have a more restricted distribution. Thus, the herein characterised FadsC appears to be a Echinoidea-specific enzyme, whereas other echinoderm classes such Asteroidea, Ophiuroidea and Holothuroidea possess an alternative desaturase herein denoted as FadsB.

## Supporting Information

S1 Fig Mass spectra of DMOX derivatives prepared from desaturation products of the *P*. *lividus* FadsA (Δ5 desaturase), namely 18:1n-13 (A), 20:4^Δ5,11,14,17^ (B) and 20:3^Δ5,11,14^ (C). Mass of 153 in red is the diagnostic mass ion that shows Δ5 desaturation and other characteristic mass ions of each product fatty acid are indicated in blue. The corresponding fragmentation patterns are also shown in each panel.
(TIFF)
